# P337: Evaluation of the level of implementation of norms and standards for quality of care in health facilities in Santiago island - Cape Verde

**DOI:** 10.1186/2047-2994-2-S1-P337

**Published:** 2013-06-20

**Authors:** A Correia, Casimiro Paula, Strong Argentina

**Affiliations:** 1 CNDC, MS, Praia, Cape Verde

## Introduction

This study falls within the framework of enhancing the performance of the Health System in Cape Verde, through the continuous improvement of the quality of care in health facilities in the country. The data collected for this study will help define a national strategy for the development of quality care systems and quality management in health facilities in the perspective of optimal management of patients in health care settings.

## Objectives

The main objective was to make an inventory of the organization and implementation of quality standards of care in health facilities, in the island of Santiago, including EmOC services.

## Methods

The standards that the level of implementation was evaluated in this study are 5. It is: Reception in EmOC services, The Hand Hygiene, Management of medical waste; disinfection of medical devices, Prevention of infection risk. The technique used for data collection in health facilities is that of Interview. It ran from a questionnaire prepared in advance and includes closed questions. Quality was judged by the average compliance levels in four levels (excellent, satisfactory, inadequate and unacceptable).

The study focused on three levels of the health pyramid.

## Results

The average compliance rate were as follows: The existence of host device in health facilities was 78%, so satisfactory; the existence device of hand hygiene in health facilities was 71%, so satisfactory; the existence of device management bio-medical waste in health facilities was 74%, so satisfactory; the existence of devices for disinfecting medical equipment in health facilities was 95%, so satisfactory; Prevention of infectious risk in health facilities was 39%, so in the limit of unacceptable.

## Disclosure of interest

None declared

